# A Comparative Study of Hospitalization Mortality Rates between General and Emergency Hospitalized Patients Using Survival Analysis

**DOI:** 10.3390/healthcare12191982

**Published:** 2024-10-04

**Authors:** Haegak Chang, Seiyoung Ryu, Ilyoung Choi, Angela Eunyoung Kwon, Jaekyeong Kim

**Affiliations:** 1 School of Management, Kyung Hee University, Seoul 02447, Republic of Korea; hkc@khu.ac.kr; 2 Department of Bigdata Analytics, Kyung Hee University, Seoul 02447, Republic of Korea; rsy22@khu.ac.kr; 3 Divison of Business Administration, Seo Kyeong University, Seoul 02713, Republic of Korea; iychoi@skuniv.ac.kr; 4 Sauder School of Business, University of British Columbia, Vancouver, BC 2053, Canada; angela.kwon@sauder.ubc.ca

**Keywords:** survival analysis, Kaplan–Meier survival analysis, cox proportional hazards model, national health insurance services cohort DB, survival period, death rate, medical data

## Abstract

Background/Objectives: In Korea’s emergency medical system, when an emergency patient arises, patients receive on-site treatment and care during transport at the pre-hospital stage, followed by inpatient treatment upon hospitalization. From the perspective of emergency patient management, it is critical to identify the high death rate of patients with certain conditions in the emergency room. Therefore, it is necessary to compare and analyze the determinants of the death rate of patients admitted via the emergency room and generally hospitalized patients. In fact, previous studies investigating determinants of survival periods or length of stay (LOS) primarily used multiple or logistic regression analyses as their main research methodology. Although medical data often exhibit censored characteristics, which are crucial for analyzing survival periods, the aforementioned methods of analysis fail to accommodate these characteristics, presenting a significant limitation. Methods:Therefore, in this study, survival analyses were performed to investigate factors affecting the dying risk of general inpatients as well as patients admitted through the emergency room. For this purpose, this study collected and analyzed the sample cohort DB for a total of four years from 2016 to 2019 provided by the Korean National Health Insurance Services (NHIS). After data preprocessing, the survival probability was estimated according to sociodemographic, patient, health checkup records, and institutional features through the Kaplan–Meier survival estimation. Then, the Cox proportional hazards models were additionally utilized for further econometric validation. Results: As a result of the analysis, in terms of the ‘city’ feature among the sociodemographic characteristics, the small and medium-sized cities exert the most influence on the death rate of general inpatients, whereas the metropolitan cities exert the most influence on the death rate of inpatients admitted through the emergency room. In terms of institution characteristics, it was found that there is a difference in determinants affecting the death rate of the two groups of study, such as the number of doctors per 100 hospital beds, the number of nurses per 100 hospital beds, the number of hospital beds, the number of surgical beds, and the number of emergency beds. Conclusions: Based on the study results, it is expected that an efficient plan for distributing limited medical resources can be established based on inpatients’ LOS.

## 1. Introduction

Despite the higher demand for emergency medical care due to various accidents, many patients could not receive proper emergency treatment, leading to an increase in the death rate. Following the COVID-19 era, there have been disruptions in the management and operation of emergency medical services since many of the medical resources, including medical staff, emergency rooms, and hospital beds, were specifically dedicated to being utilized in the COVID-19 emergency medical centers [1]. 

In Korea’s emergency medical system, upon the emergence of an emergency patient, treatment is administered on-site and during transport at the pre-hospital stage, followed by inpatient care at the hospital stage [2,3]. As such, the role of emergency rooms in this emergency medical system is becoming increasingly important. Generally, under Article 31 of the Emergency Medical Service Act, emergency rooms are designed to provide emergency care and other medical tasks and are staffed 24 h by specialist physicians who provide efficient and prompt treatments. In fact, emergency rooms have confronted the issue of overcrowding due to an influx of visits by both patients who require immediate care and those with non-emergency conditions.

Overcrowding in emergency rooms is a phenomenon resulting from the lack of medical resources and treatments, which are insufficient to meet the demands of emergency care. This phenomenon leads to several negative impacts, including a prolonged waiting time in the emergency room, an increase in the death rate due to ambulance delay, an inability to prepare for major disasters, and a decline in the quality of emergency medical services due to the allocation of resources to non-emergency patients. To address such issues, there have been many studies suggesting causes and solutions of emergency room overcrowding [4,5,6,7]. Despite the foundation of such issues stemming from knowing the determinants of patients’ death in the emergency room, it is often neglected from the perspective of emergency management and treatment. In fact, the post-traumatic death rate in 2019 in Korea is preventable by 15.7%, which is not significantly different from the rates in other developed countries [8]. It has been reported that the post-traumatic death rate could be reduced to below 10% with an augmented emergency medical system [9]. Therefore, to reduce the preventable post-traumatic death rate, it is vital to identify determinants affecting the surviving period of inpatients admitted through the emergency room. However, if the study observes the time of death for inpatients admitted through the emergency room only, it remains ambiguous whether one survived during the observation period, and, thus, preventing full identification of their exact survival time. Moreover, if an inpatient died due to other factors besides one’s post-traumatic conditions, it is impossible to know one’s survival time. Therefore, the aforementioned types of records regarding patients’ death all correspond to the censored data, which is a significant characteristic to be considered prior to conducting data analyses. In fact, multivariate and logistic regression analyses do not account for the censored nature of data, and, thus, this places a limitation to the investigation of determinants affecting the surviving period of patients admitted through the emergency room. It is necessary to minimize the inappropriate use of emergency room treatments by non-emergency patients because care consists of emergency room treatment and inpatient care at the hospital stage of the emergency medical system. In particular, considering an increase in the death rate within the emergency room department in Korea, it is urgent to carefully identify the determinants of survival time for ER-admitted patients. For this purpose, this study aims to identify factors influencing the survival time of general inpatients and those admitted through the emergency room and to compare the results between the two groups of patients. Since data regarding an individual’s death are the censored data, it is important to consider the survival time prior to analyzing its determinants. Therefore, we decided to utilize survival analysis as the main methodological tool for our research. In fact, survival analysis enables us to statistically analyze not only the duration until the death of inpatients admitted in the emergency room but also the determinants of the survival time [10,11]. This study aims to compare the survival probabilities over time for ER-admitted patients and general inpatients using the non-parametric statistical method of the Kaplan–Meier survival estimation. Then, using the semi-parametric statistical method of the Cox proportional hazards model, this study seeks to analyze and compare features influencing death rates between inpatients admitted in the emergency room and general inpatients. The Cox proportional hazards model is expressed by the hazard function. That is, the hazard function can be interpreted as the risk of death at time. Therefore, the measure of effect in the Cox model is the hazard rate, i.e., the risk of death given that the patient survived until a specific time. We use the cohort database of the National Health Insurance Corporation of Korea. The data include information on the date of death of emergency patients and general patients after hospitalization, but there is no information on whether the patient was discharged after full recovery or if transferred to another hospital. Therefore, we assume death as a terminal event and perform survival analysis.

In terms of features utilized in this study, we referred to the extant literature and selected ones from the cohort DB provided by the Korean NHIS. The data analysis was conducted in a virtual environment of NHIS using the R statistical program (version 3.7.6). Since this study identifies the main determinants affecting the dying risk of inpatients, we expect medical institutions to allocate medical resources in an effective manner. We also expect guidelines to be established to address emergency room overcrowding, based on information about patients prioritized for emergency care.

## 2. Theoretical Background

### 2.1. Emergency Medical Services (EMS)

**Figure 1:**
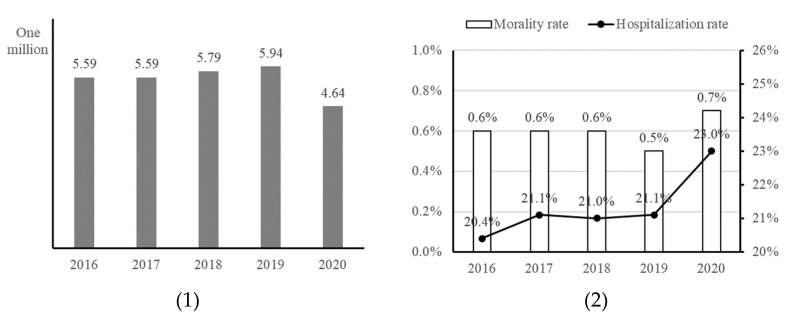
Current state of EMS in Korea (Source: KOSIS (Korean Statistical Informational Service, Statistics of EMS, 21 November 2022)). The two represent (1) use of emergency room, and (2) admission and death rate in emergency (from left to right).

The emergency medical services (EMS) encompass actions taken for patients from the onset of an emergency until they recover from life-threatening conditions or are alleviated from physical and psychological harm [12]. As a part of public goods, these services include consultation, rescue, transport, emergency treatment, and medical care [13]. As shown in Figure 1(1), the number of patients using emergency rooms increased from 5.59 million in 2016 and 2017 and 5.79 million in 2018 to 5.94 million in 2019, which then decreased to 4.64 million in 2020. The admission and death rates in the emergency room are shown in Figure 1(2). The death rates gradually increased from 0.6% in 2016, 0.6% in 2017, 0.6% in 2018, 0.5% in 2019, and 0.7% in 2020. The admission rates also increased from 20.4% in 2016, 21.1% in 2017, 21.0% in 2018, 21.1% in 2019, and 23.0% in 2020.

Despite the increasing trends in death and admission rates of emergency patients, it is difficult to find existing studies examining the relationship of death and admission rates of emergency patients with their survival time. In fact, most existing research related to emergency room is often focused on ER overcrowding; however, it is necessary to analyze the survival time for ER-admitted patients prior to scrutinizing the death and admission rates of these individuals.

### 2.2. Survival Analysis

**Figure 2:**
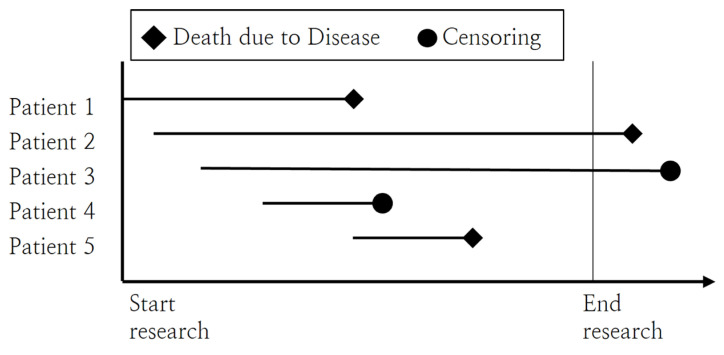
Example of censored data.

Survival analysis is a method widely used in the fields of biology and medicine, which utilizes censored data containing information, such as patients’ survival and death, and assesses differences in the elapsed time to an event of interest [11,14,15,16]. Here, censored data refers to data with unknown occurrence of an event from the beginning of the study to the end. For instance, when observing the time of death among patients as in Figure 2, the characteristics for each data set are as follows. The data for patients 1 and 5 fall under complete data, while patient 2, 3, and 4 correspond to censored data. In particular, the data for patient 4 is considered censored because the cause of death was unrelated to the aggravated disease.

Since survival analysis is a statistical approach that estimates the survival time between two events of interest, it is explicitly different from other approaches such as regression and logistic regression, as demonstrated in Table 1. While linear regression considers time as a dependent variable, it is limited by not accounting for the presence of censored data. On the other hand, logistic regression can only include an event, such as whether one has died or has been hospitalized, as a dependent variable, but it cannot consider time in its analysis.

In fact, survival analysis can be conducted using three types of methods: non-parametric, semi-parametric, and parametric methods. First, a non-parametric method does not require an assumption that the data follow a certain probability distribution. Second, a semi-parametric method still does not require an assumption regarding data distribution yet estimates regression coefficients. Lastly, a parametric method carries an assumption that the data follows a distribution, such as the Weibull distribution, with respect to survival time, *t*.

Among non-parametric methods, there are the Kaplan–Meier estimation analysis and the log-rank test. The Kaplan–Meier estimation assumes that events are to occur independently of one another and calculates survival probabilities from one interval to the next under the assumption that censoring is independent of the survival time [17]. These probabilities can be illustrated in a survival plot [17]. The log-rank test compares the time-to-event distributions across two or more independent groups, utilizing a chi-squared test of the time occurrence between the observed and expected counts. This test is particularly used to validate the null hypothesis that no significant difference exists in the survival curves between the groups being compared.

Here, Table 2 demonstrates the existing literature that utilized survival analysis for research purposes across various fields of study. In fact, there is one semi-parametric method, which is the Cox proportional hazards model. This model is a multivariate regression method that tests the significance of various predictors relevant to time and processes the censored data, assuming that there is a log-linear relationship between the survival function and the variables [18]. Having acknowledged that the data used in this study do not satisfy a certain distribution over time, such as the Weibull distribution, we decided to utilize the Kaplan–Meier estimation and the Cox proportional hazards model. In other words, this study aims to estimate and compare the survival time of general inpatients and patients admitted through the emergency room using the Korean NHIS cohort DB based on the Kaplan–Meier survival analyses.

## 3. Research Methodology

### 3.1. Research Framework

**Figure 3:**
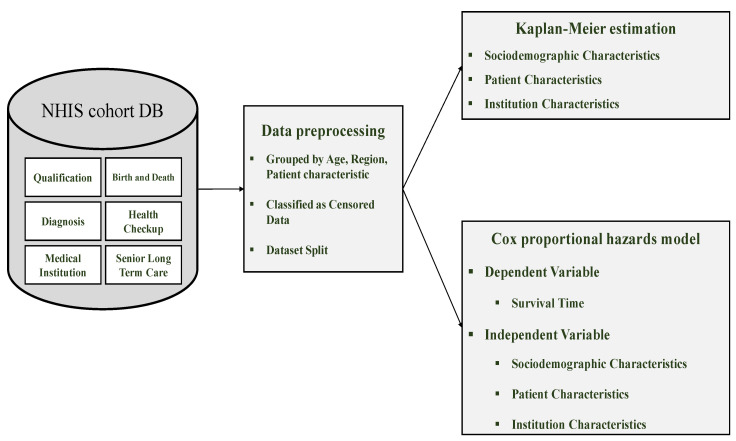
Research framework for investigating determinants of survival time.

The purpose of this study is to identify factors affecting the survival time of general inpatients and inpatients admitted through the emergency room, separately, and to compare the results. General inpatients, in this study, refer to those who were hospitalized without the emergency room transport. As demonstrated in Figure 3, we conducted our research in three phases: data collection, data preprocessing, and survival analysis.

During the data collection phase, we collected the Korean National Health Insurance Service (NHIS) cohort DB. For data preprocessing, we grouped the features, classified the censored data, and divided the subjects into general inpatients and those admitted through the emergency room. Lastly, for survival analyses, we investigated the main determinants of survival time for each group of subjects using both the Kaplan–Meier estimation and the Cox proportional hazards model.

### 3.2. Data Collection

To identify determinants affecting the dying risk of general inpatients and that of inpatients admitted through the emergency room, this study utilized the four-year health checkup cohort DB from the year 2016 to 2019 provided by the Korean National Health Insurance Services. This cohort DB is a sample study DB established in January 2013 based on the health examination records DB, encompassing approximately one million (2% of the total population in Korea) records of patients without violating the privacy terms. This cohort DB is the latest data provided by the Korea Health Insurance Service.

Such DB is largely composed of six different tables as follows: qualification, birth and death, diagnosis, health checkup, medical institution, and senior long-term care (see Table 3).

Referring to prior studies on investigating determinants of survival time, we selected four tables out of the displayed tables in the cohort DB for the purpose of our study: qualification, birth and death, diagnosis, and medical institution. As presented in Table 4, a total of 18 variables were used in this study, where 12 of them were classified into either of the sociodemographic, patient, and institution tables for analyzing the determinants of survival time.

First, from the ‘qualification’ and ‘birth and death’ tables, we utilized the subjects’ sociodemographic and personal information. Among the sociodemographic features, information such as one’s gender, age, and region was included. Among the patient features, information such as one’s type of health insurance, income quantile, and the severity of disability was included. We used the information regarding one’s death from the ‘birth and death’ table as a dependent variable of our analyses. Second, from the ‘Diagnosis’ table, we utilized information pertaining to whether one has been hospitalized and also the admission route of hospitalization in order to compare the survival time between general inpatients and those admitted through the emergency room. We also used information regarding the diagnostic results as the censored data. Third, we used various information on institutions from the ‘Medical Institution’ table. This table includes features, including the types of institutions that one has been admitted, the number of doctors and nurses, and the number of hospital beds.

### 3.3. Data Preprocessing

This study conducted survival analyses on subjects with hospitalized records between 2016 and 2019 from the sample cohort DB provided by the Korean NHIS. Data preprocessing prior to conducting survival analysis involved eliminating the missing values, merging between tables, and extracting values for each feature followed by identifying the censored data. The details of data preprocessing are presented in Table 5.

First, the process of eliminating missing values was completed using the features corresponding to the income quantile, the severity of disability, diagnostic results, and the date of death. In fact, missing values found within the income quantile and diagnostic results category were eliminated prior to analysis, while ones for the severity of disability were replaced with ‘no inclusion (normal)’. Missing values within the date of death feature were instead indicated as ‘survived’, implying that the patients had not passed away. Second, we only extracted the records for top general hospitals and general hospitals using the codes indicating a type of institution. Next, we utilized common features, such as the standard year code and the code for interlinking the tables to merge the four tables of our research interest. The remaining number of records after merging is 25,722,085. Third, we completed feature preprocessing by, for instance, grouping the variables. In terms of age, since the year of birth was provided by the NHIS data, it was converted to age as of the base year, which was then grouped into ten-year units. For region, we referred to the extant literature [25] to classify the variables into Seoul, metropolitan cities (Busan, Daegu, Daejeon, Incheon, Gwangju, Ulsan), and the others as the small and medium-sized cities. Among the patient characteristics, for insurance status, we regrouped the existing six categories of the feature into three categories: medical insurees, regional insurance, and workplace insurance. For income quantiles, we reclassified the existing ten different quantiles of income into 0 quantile, 1–3 quantiles, 4–7 quantiles, and 8–10 quantiles. For institutional characteristics, considering the size of medical institutions used in this study, we set the number of doctors and nurses by 100 hospital beds, which were then grouped into four separate groups. The same procedure was applied for the number of hospital, surgical, and emergency beds. Fourth, censored data, which are not conventionally considered in other analytical methods, can be utilized in survival analysis. Therefore, this study accounted for subjects who had not died by the last date of treatment—that is, subjects whose current survival status is unknown—to be classified as censored data.

## 4. Results

This study conducted survival analyses on subjects with hospitalized records between 2016 and 2019 from the sample cohort DB provided by the Korean NHIS. Data preprocessing prior to conducting survival analysis involved eliminating the missing values, the merging between tables, and extracting values for each feature followed by identifying the censored data.

### 4.1. Determinants of Survival Time among General Inpatients

For the purpose of finding determinants of survival time among general inpatients and those admitted through the emergency room, we first conducted survival analysis on general inpatients. A total of 3,228,933 records was used, and 12 variables were investigated to check which of them affect the risk of death.

#### 4.1.1. Characteristics of General Inpatients

Table 6 presents the characteristics of general inpatients. The gender distribution of the study subjects is 50.13% male and 49.87% female. The age distribution is as follows: 11.66% under 29, 5.62% between 30 and 39, 9.42% between 40 and 49, 17% between 50 and 59, 19.18% between 60 and 69, 21.21% between 70 and 79, 13.86% between 80 and 89, and 2.05% above 90. For region, the result shows 21% in Seoul, 31% in metropolitan cities, and 47% in other small and medium-sized cities. For insurance status, 10% are medical insurees, 29% are those with regional insurance, and 61% are those with workplace insurance. For income quantiles, 10%, 20%, 31%, and 38% are 0, 1–3, 4–7, and 8–10 quantiles, respectively. For the severity of disability, individuals with normal, mild, and severe symptoms are 79%, 11%, and 10%, respectively. There are 70% general hospitals and 30% top general hospitals. For other features, such as the number of doctors per 100 hospital beds, they were previously divided into four groups, and, thus, there is 25% for each category.

#### 4.1.2. Kaplan–Meier Estimation (General Inpatients)

This study utilizes the Kaplan–Meier estimation to analyze how the sociodemographic, patient, health checkup, and institution features affect LOS for inpatients over time.

**Figure 4:**
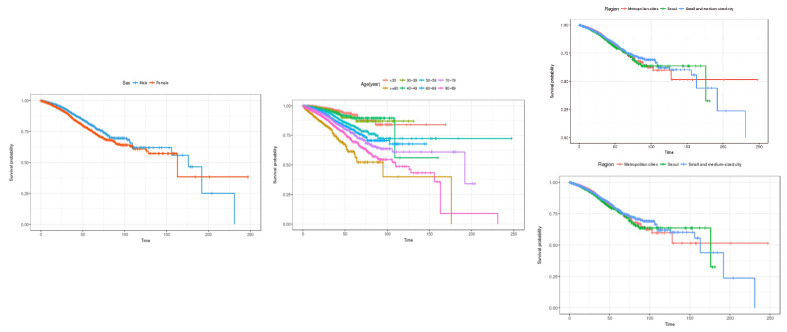
Kaplan–Meier survival curves by socio-demographic characteristics (general inpatients). The three charts represent (1) sex, (2) age, and (3) region (from left to right).

First, the estimates for the features responsible for sociodemographic information, including gender, age, and city/province are shown in Figure 4. In the case of gender, the survival rate for men appears to be better than for women up to about 180 days, but after that, the survival rate for women appears to be higher. In terms of age, the survival rate gradually decreases from those in their 30s to those in their 90s or older. Lastly, in the case of regional areas, the survival rate in small and medium-sized cities appears to be high up to about 100 days, but the survival rate decreases rapidly after that. On the other hand, the survival rate in Seoul was the highest from about 100 to 180 days, and the survival rate in metropolitan cities was high after about 180 days.

**Figure 5:**
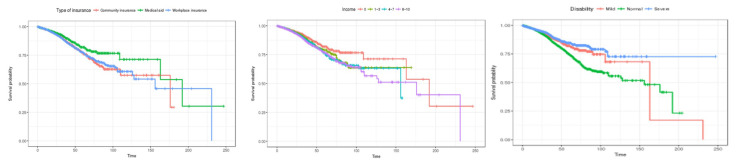
Kaplan–Meier survival curves by patient characteristic (general inpatients). The three charts represent (1) type of insurance, (2) income, and (3) disability (from left to right).

Second, patient characteristics, such as health insurance subscriber classification, income bracket, and disability severity results, are shown in Figure 5. In the case of health insurance subscriber types, the survival rate of medical benefit recipients was the highest, and the survival rate tended to decrease in that order: local subscribers, and employer subscribers. In terms of income quintile, the 0th quintile showed the highest survival rate, and the survival rate decreased in that order: 8th to 10th quintile, 4th to 7th quintile, and 1st to 3rd quintile. In terms of disability severity, the survival rate was found to be high for severely ill patients, followed by the highest survival rate for mild patients. However, after about 160 days, the survival rate of patients with dysentery is rapidly decreasing, and the survival rate of normal patients (not applicable) appears to be higher.

**Figure 6:**
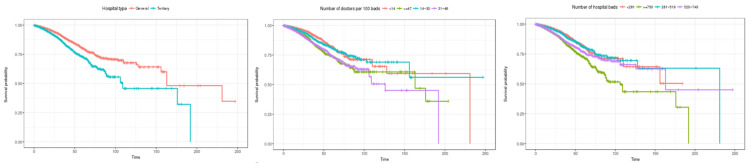
Kaplan–Meier survival curves by institution characteristic (general inpatients). The three charts represent (1) hospital type, (2) number of doctors per 100 beds, and (3) number of hospital beds (from left to right).

Third, the Kaplan–Meier survival curve by characteristics of medical institutions is shown in Figure 6. Looking at the survival rate by hospital type, it is estimated that the overall survival rate of general hospitals is higher than that of tertiary general hospitals. In terms of the number of doctors per 100 beds, the survival rate is estimated to be lowest in the order of 14 or less in the initial stage of hospitalization, followed by 14 to 30, 47 or more, and 31 to 46. Looking at the number of hospital beds, the survival rate is estimated to be high initially in the order of 281 or less, 281 to 519, 520 to 749, and 750 or more. This shows that, overall, the survival rate is estimated to be lower in larger hospitals. This is believed to be because the period of hospitalization in larger hospitals is limited, meaning there is a lot of censored data.

#### 4.1.3. Cox Proportional Hazards Model (General Inpatients)

**Figure 7:**
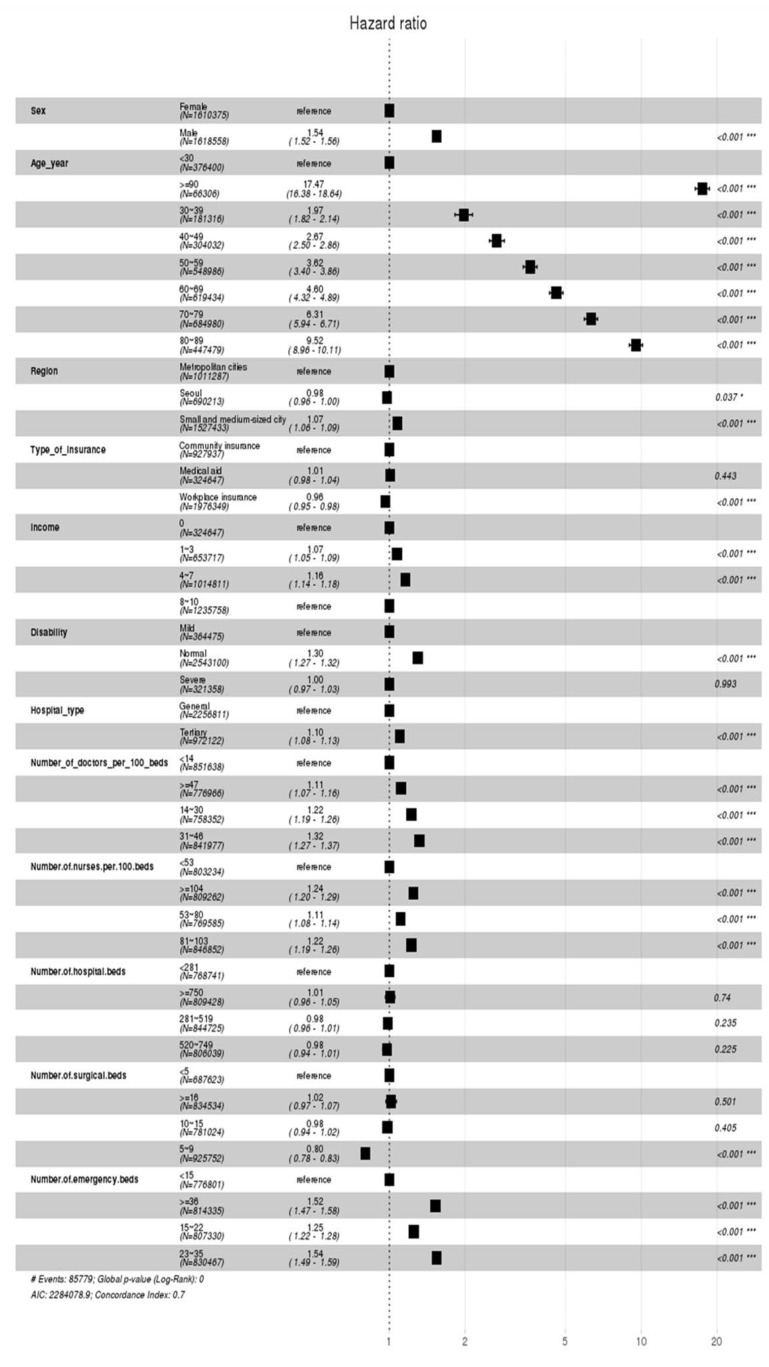
Cox proportional hazards model indicating the hazard ratio of the general inpatients. (* *p* < 0.05, *** *p* < 0.001).

The Cox proportional hazards model results for determinants affecting the dying risk of general inpatients are shown in Figure 7. First, in terms of sociodemographic characteristics, it was found that the death rate for men is 1.54 times higher than for women. The death rate increases from under 30s to the 90s, with those over 90 having a death rate 17.47 times higher than those under 30. For region, compared to the metropolitan cities, the death rate in the small or medium-sized cities increases by 1.07 times, while it decreases by 0.98 times in Seoul. Second, among patient characteristics, the results for the type of insurance showed that the death rate of medical insurees increases by 1.01 times than that of those with regional insurance, while it decreases for those with workplace insurance to 0.96 times, indicating a lesser impact on mortality. In terms of income quantiles, the impact on death rate for patients in the 4–7 quantiles increases by 1.16 times of those in the 0th quantile. For the severity of disability, the impact on the death rate is 1.3 times higher for normal patients compared to those with mild disabilities. Third, the death rate within the top general hospitals is 1.10 times higher than that within the general hospitals. For the number of doctors per 100 hospital beds, the death rates for 31–48, 14–30, and above 47 doctors are 1.32, 1.22, and 1.11 times the death rate below 14 doctors, respectively. For the number of nurses per 100 hospital beds, it was found that compared to a group with fewer than 53 nurses, the death rates are higher in the following order: 1.24 times for the group over 104 nurses, 1.22 times for the group over 81 to 103 nurses, and then for the group between 53 and 80 nurses. In fact, there is no statistically significant difference in the results between different groups of the hospital beds. However, for the number of surgical beds, it was found that hospitals with five to nine beds have a death rate 0.8 times lower than hospitals with fewer than five beds, while there is no statistically significant differences in those with eighteen or more beds and ten to fifteen beds. Lastly, hospitals with the fewest emergency beds, which are less than 15, have the lowest death rate, while those with 23–35, more than 38, and 15–22 beds have the death rates that are 1.54, 1.52, and 1.25 times higher, respectively.

### 4.2. Determinants of Survival Time among Inpatients Admitted through Emergency Room

#### 4.2.1. Characteristics of Inpatients Admitted through the Emergency Room

Table 7 presents the characteristics of inpatients admitted through emergency room. The gender distribution of the study subjects is 51.26% male and 48.74% female. The age distribution is as follows: 12.88% under 29, 5.72% between 30 and 39, 8.42% between 40 and 49, 14.47% between 50 and 59, 16.76% between 60 and 69, 21.33% between 70 and 79, 17.46% between 80 and 89, and 2.94% above 90. For region, the result shows 23.15% in Seoul, 25.54% in metropolitan cities, and 51.31% in other small and medium-sized cities. For insurance status, 9.88% are medical insurees, 28.78% are those with regional insurance, and 61.34% are those with workplace insurance. For income quantiles, 9.88%, 20.17%, 30.61% and 39.35% are 0, 1–3, 4–7, and 8–10 quantiles, respectively. For the severity of disability, individuals with normal, mild, and severe symptoms are 77.65%, 11.46%, and 10.96%, respectively. There are 61.39% general hospitals and 38.61% top general hospitals. For other features, such as the number of doctors per 100 hospital beds, they were recategorized into four groups during data preprocessing. For the number of doctors per 100 hospital beds, 12.13%, 22.05%, 35.72%, and 30.10% are institutions with below 13, 14–30, 31–46, and above 47 doctors per 100 hospital beds, respectively. For the number of nurses per 100 hospital beds, 12.54%, 20.40%, 35.04%, and 32.02% are institutions with 52, 53–80, 81–103, and above 104 nurses per 100 hospital beds, respectively. For the number of hospital beds, 12.43%, 20.65%, 35.27%, and 31.64% are institutions with below 280, 281–519, 520~749, and above 750 hospital beds, respectively. For the number of surgical beds, 11.71%, 21.86%, 33.41%, and 33.02% are institutions with below 4, 5–9, 10–15, and above 16 surgical beds, respectively. Lastly, for the number of emergency beds, 10.34%, 21.85%, 33.24%, and 34.57% are institutions with below 15, 15–22, 23–35, and above 36 surgical beds, respectively.

#### 4.2.2. Kaplan–Meier Estimation (Inpatients Admitted through the Emergency Room)

**Figure 8:**
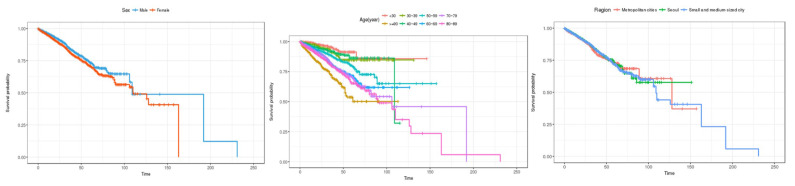
Kaplan–Meier survival curves by socio-demographic characteristics (inpatients admitted through the emergency room). The three charts represent (1) sex, (2) age, and (3) region (from left to right).

To estimate the survival rate for patients admitted to the emergency room over time, Kaplan–Meier estimation was performed by sociodemographic characteristics, patient characteristics, and medical institution characteristics. First, the survival probability estimation results for socio-demographic characteristics, such as gender, age, and region, are shown in Figure 8. In the case of gender, it is estimated that men have a higher survival probability than women in the early days of hospital stay, but after 125 days of hospital stay, the survival rate of men is estimated to be better than that of women.

In terms of age, the overall survival rate shows a gradual decreasing trend from those in their 30s to those in their 90s, but after the 100th day of hospitalization, the survival rate for those in their 40s is estimated to be lower than that for those in their 90s or older. In the case of regions, there appears to be no difference in the survival rate in the early days of hospitalization, but after 125 days of hospitalization, the survival rate is estimated to be the best in metropolitan cities, followed by Seoul and small and medium-sized cities.

**Figure 9:**
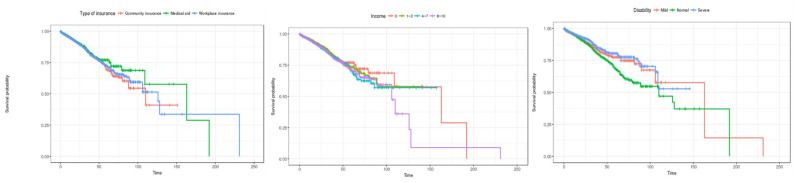
Kaplan–Meier survival curves by patient characteristics (inpatients admitted through the emergency room). The three charts represent (1) type of insurance, (2) incom, and (3) disability (from left to right).

Second, patient characteristics, such as health insurance subscriber type, income bracket, and disability severity, are shown in Figure 9. In the case of health insurance subscriber types, there was no difference in survival rate at the beginning of the length of stay, but after about 50 days, the survival rate was highest for medical benefit recipients, and the survival probability tended to decrease in the order of employer subscribers and local subscribers. In the case of income brackets, it is estimated that there is no difference in survival rate in the early stages of hospital stay. In the 8th to 10th percentiles, the survival rate showed a sharp decline after 100 days of hospitalization. In the case of disability severity, it is estimated that the survival rate is high in the order of severe, mild, and normal patients at the beginning of the length of stay. However, at 125 days, contrary to general hospitalized patients, the survival rate was highest for mild patients, and the survival rate decreased in that order for severe patients and then normal patients. It is estimated that the survival rate of mildly ill patients will decline sharply after 150 days.

**Figure 10:**
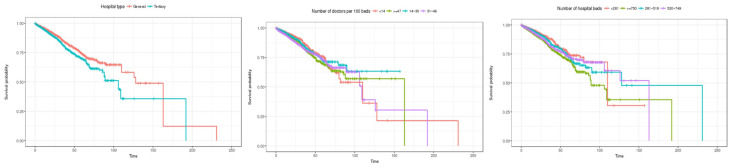
Kaplan–Meier survival curves by institution characteristic (inpatients admitted through the emergency room). The three charts represent (1) hospital type, (2) number of doctors per 100 beds, and (3) number of hospital beds (from left to right).

Third, the Kaplan–Meier survival curve by characteristics of medical institutions is shown in Figure 10. Looking at the hospital type, it is estimated that the survival rate is higher in general hospitals than in tertiary general hospitals. In terms of the number of doctors per 100 beds, it was initially estimated that hospitals with less than 14 doctors (the group with the fewest doctors) had the highest survival rate, but the survival rate was found to decline sharply after about 60 days. In terms of the number of hospitalized beds, the survival rate was estimated to be high in the following order: less than 281 beds, 281 to 519, 520 to 749, and more than 750 beds until the length of stay was about 60 days. However, at about 110 days, the survival rate of hospitals with fewer than 281 beds was found to decline sharply.

#### 4.2.3. Cox Proportional Hazards Model (Inpatients Admitted through the Emergency Room)

**Figure 11:**
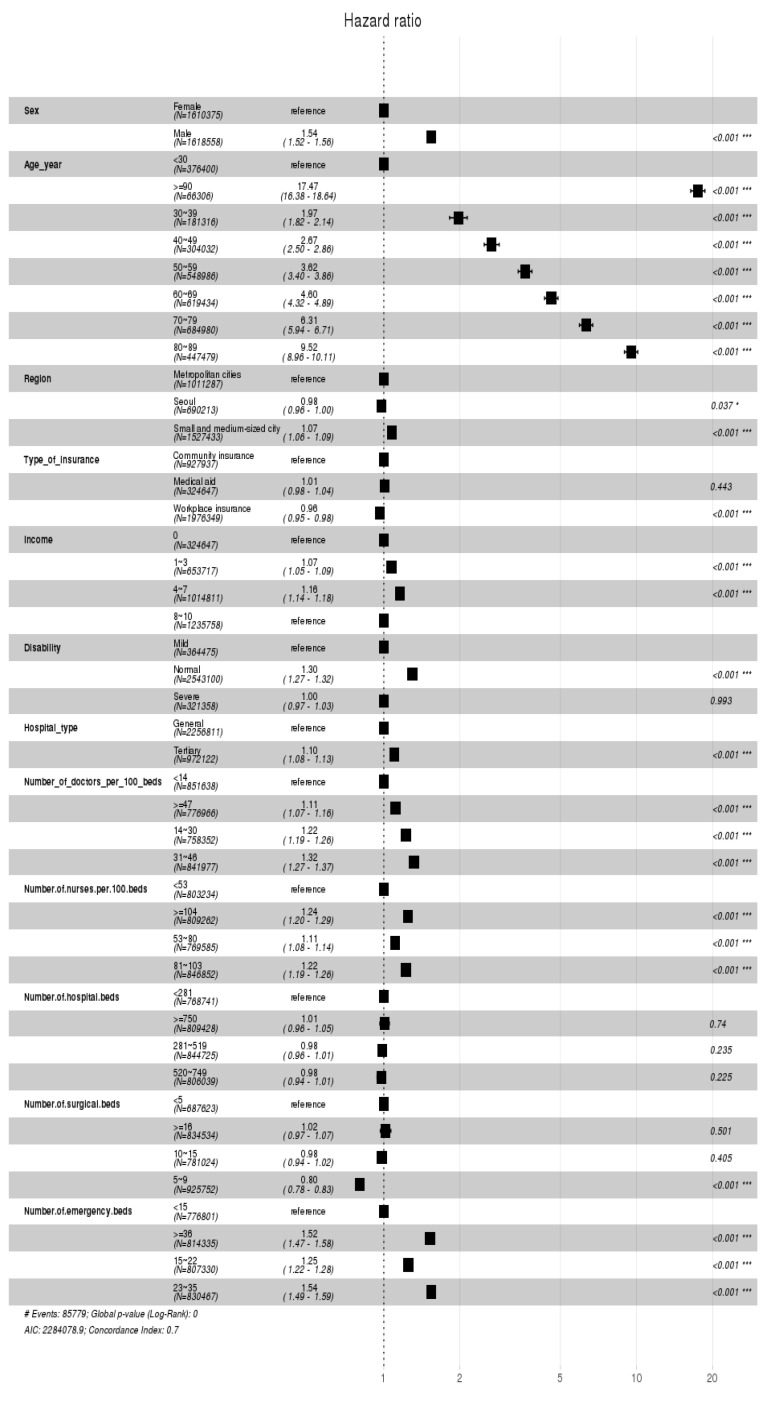
Cox proportional hazards model indicating the hazard ratio of the inpatients admitted through the emergency room. (* *p* < 0.05, *** *p* < 0.001).

The Cox proportional hazards model demonstrates the determinants affecting the dying risk of inpatients admitted through the emergency room, as shown in Figure 11.

First, in terms of sociodemographic characteristics, it was found that the death rate for men is 1.43 times higher than for women. The death rate increases from under 30s to the 90s, with those over 90 having a death rate 12.20 times higher than those under 30. For region, compared to the metropolitan cities, the death rate in the small or medium-sized cities decreases by 0.96 times, while it also decreases by 0.95 times in Seoul. Second, among patient characteristics, the results for the type of insurance showed that the death rate of medical insurees increases by 1.07 times than that of those with regional insurance, while it decreases for those with workplace insurance to 0.93 times, indicating a lesser impact on mortality. In terms of income quantiles, the impact on death rate for patients in the 4–7 quantiles increases by 1.16 times to those in the 0th quantile, while its impact is the same between the 0th quantile and the 8–10 quantiles. Similarly, for the severity of disability, the impact on the death rate is 1.22 times higher for normal patients than for patients with mild disabilities, while the death rate for patients with severe disabilities decreases by 0.94 times than that for patients with mild disabilities. Third, the death rate within the top general hospitals is 1.06 times higher than that within the general hospitals. For the number of doctors per 100 hospital beds, the death rates for 14–30 and 31–46 doctors are 1.21 and 1.19 times the death rate below 14 doctors, respectively. However, there is no statistically significant difference in death rates between the institutions with fewer than 14 doctors and ones with more than 47 doctors. For the number of nurses per 100 hospital beds, the death rates for 281–519 and for more than 750 nurses are 1.10 and 1.07 times the death rate below 53 nurses, respectively. However, there is no statistically significant difference in death rates between the institutions with fewer than 53 nurses and ones with 520–749 nurses.

For the number of hospital beds, institutions with the third highest number of beds (281–519) and those with 750 beds have increased death rates of 1.10 and 1.07 times higher, respectively, than those with the fewest beds (below 281). However, there is no significant difference in the death rates between institutions having lower than 281 beds and those with 520–749 beds. For the number of surgical beds, compared to institutions with the fewest number of surgical beds (below 5), those with the highest number of surgical beds (above 16) and those with 10–15 surgical beds have the death rate of 1.13 and 1.09 times higher, respectively. In contrast, those with 5–9 surgical beds demonstrated the decreased death rate that is 0.80 times than that of those with the fewest number of surgical beds. Lastly, in terms of the number of emergency beds, those with above 36, 23–35, and 15–22 emergency beds have increased death rates of 1.40, 1.34, and 1.16 times higher, respectively, than those with the fewest number of emergency beds (below 15).

### 4.3. Summarized Results and Implication

Table 8 illustrates the summarized results of this study. In fact, there is no significant difference in determinants of the death rate between the two groups of study. However, in terms of the ‘city’ feature among the sociodemographic characteristics, the small and medium-sized city exerts the most influence on the death rate of general inpatients, whereas the metropolitan city exerts the most influence on the death rate of inpatients admitted through the emergency room. In terms of institution characteristics, it was found that there is a difference in determinants affecting the death rate of the two groups of study, such as the number of doctors per 100 hospital beds, the number of nurses per 100 hospital beds, the number of hospital beds, the number of surgical beds, and the number of emergency beds.

The theoretical implications of this study are as follows. This study is the pioneering research in analyzing determinants affecting the death rate of general inpatients as well as that of inpatients admitted through the emergency room using survival analyses. Therefore, we first utilized the Kaplan–Meier survival estimation to take a closer look at the change in survival probability of inpatients depending on their sociodemographic, patient, and institutional characteristics. We also incorporated the Cox proportional hazards model to investigate not only the statistically significant features from sociodemographic, patient, and institutional characteristics that influence the death rate of inpatients but also the extent to which each feature affects mortality.

The practical implications of this study are as follows. Although Korea has a multiple regional emergency medical centers across the country (Seoul: 27, Incheon: 10, Busan: 8, Daegu: 5, Daejeon: 4, Ulsan: 1, Gwangju: 5, Gyeonggi: 22, Gyeongbuk: 6, Gyeongnam: 6, Chungbuk: 4, Chungnam: 8, Jeonbuk: 8, Jeonnam: 2, Gangwon: 4, Jeju: 4), the survival probability of emergency room patients within the metropolitan cities is found to be the lowest. This is most likely due to the inadequate initial treatment and procedures for critically ill emergency patients at the regional emergency medical centers.

Furthermore, the emergency medical expense system is structured in a way that the more patients visit the emergency room, the more revenue is generated, regardless of the investment towards the emergency room or its quality of care. Therefore, to solve such issues, it is of importance to expand regional emergency centers that specialize in the professional treatment and care of critically ill emergency patients, and to reinforce the expense system that can induce the enhancement in the quality of emergency care. Lastly, it is necessary to secure an intermediary organization that can handle medical accidents that may occur during emergency treatments.

## 5. Conclusions

### 5.1. Research Implications

Considering an increase in the death of patients within the emergency room department, it is of necessity to identify the determinants of survival time among inpatients admitted through the emergency room. Therefore, our goal was to conduct a comparative study between general inpatients and those admitted through the emergency room, using survival analyses to identify the determinants of survival time.

In fact, the results reveal that there is not much difference in the death rate between the two groups of interest. However, for the regional variable among sociodemographic features, it was found that the small and medium-sized cities exert the most influence on the death rate among general inpatients, while the metropolitan cities exert the most influence on the death rate among those admitted through the emergency room. Among institutional features, the number of doctors per 100 hospital beds, the number of nurses per 100 hospital beds, the number of hospital beds, the number of surgical beds, and the number of emergency beds were found to affect the death rate of the two groups of study subjects differently.

Many previous studies utilized multiple or logistic regression analyses as their main research methodology. However, multiple regression analysis requires basic assumptions to be met, including linearity, independence, equal variance, normality, and the absence of multicollinearity. Logistic regression analysis, on the other hand, requires assumptions, such as the linearity of the logit, the independence of the error term, and the absence of multicollinearity. Although medical data often exhibit censored characteristics, these two methods fail to accommodate them, both presenting a significant limitation.

Therefore, this study conducted survival analyses to analyze the factors affecting the dying risk of general inpatients and those admitted through the emergency room. For this purpose, we measured the probability of survival as well as that of hospitalization depending on the sociodemographic, patient, health checkup, and institutional features using the Kaplan–Meier estimation. However, the Kaplan–Meier survival estimation has a limitation in that it cannot control for factors outside of those under analysis. Therefore, we also incorporated the Cox proportional hazards models as an additional econometric method to validate the results by controlling for other factors. Since both the Kaplan–Meier survival analysis and the Cox proportional hazards model do not require assumptions regarding the data distribution, these two methods are suitable for analyzing the determinants of survival time using the medical data.

In this study, we conducted survival analyses to compare and analyze the two subject groups: general inpatients and inpatients admitted through the emergency room. It is expected that a plan for the efficient allocation of limited medical resources can be established based on our research findings.

### 5.2. Limitations and Future Directions

The limitations of this study are as follows. Although there are various features affecting the dying risk of patients, such as sociodemographic and disease-specific features, this study is limited in that we only incorporated the sociodemographic, patient, and institutional features under analyses. Therefore, future studies are to encompass a broader scale of features from various aspects.

This study separately analyzes the two patient populations (general inpatients and inpatients admitted through the ER). An analysis that incorporates both patient populations using the Cox model would enable an assessment of whether the hazard or risk (death rate) differs between the patient populations after adjusting for all the factors considered in this paper.

Moreover, we conducted survival analyses on individuals who were either general inpatients or inpatients who were admitted through the emergency room. However, it is likely that survival time varies depending on a patient’s main diagnosis. Therefore, it is highly recommended that future research should rigorously scrutinize and compare the survival times of patients across different diagnoses.

Lastly, this study conducted survival analyses based on the four-year cohort DB provided by the Korean NHIS from the years 2016 to 2019 encompassing tables of qualification, birth and death, diagnosis, health checkup, institution, and senior long-term care characteristics. However, the Korean NHIS further provides key information regarding, for instance, medical treatment (code for drugs, treatment code, main diagnosis code, days of hospitalization, etc.) and prescription details (drug ingredient code, dosage per administration, daily dosage, total days of administration, unit price, total cost, etc.). Therefore, future research should utilize the aforementioned information into their analyses.

## Figures and Tables

**Table 1:** Comparison of research methodologies for analyzing survival period.

Category	Characteristics	Limitation
Linear Regression	Dependent variable: time	Cannot consider the presence of censored data
Logistic Regression	Dependent variable: event	Cannot consider time
Survival Analysis	Can consider both time and the presence of censored data	

**Table 2:** Extant literature using survival analysis.

Researchers	Research Methodology	Research Subjects	Research Purpose
[19]	Log-rank test Cox proportional hazards model	Resort facilities in Spain	To identify financial and non-financial factors affecting the survival of resort facilities in Spain
[20]	Log-rank test Cox proportional hazards model	Resort facilities in Spain	To investigate factors affecting the survival of resort facilities in Spain
[21]	Cox proportional hazards model	Companies undergoing financial distress	To investigate how restraints to corruptions and financial ratios affect the survival of companies undergoing financial distress
[22]	Cox proportional hazards model	Companies undergoing financial distress	To investigate how factors including corporate governance, financial ratios, and political risk affect the company’s survival
[23]	Kaplan–Meier estimation Cox proportional hazards model	Pancreatic cancer patients	To estimate the prognostic effect of the established cancer hallmark genes in various cancer types
[24]	Kaplan–Meier estimation	Playtimes in game	To propose new methods to measure game playtimes

**Table 3:** Description of health checkup cohort DB.

Table	Description
Qualification	Includes socio-demographic information (gender, age, residential area, income range, insurance type) of a health checkup examinee (excluding foreigners) or information about the matter of death.
Birth and Death	Includes information on subjects whose death has been verified, linked with birth and the cause of death information provided by Statistics Korea.
Diagnosis	Includes medical records (main diagnosis information, prescription history, cost-related information, admission records, the department of treatment, etc.); consists of ten DB partitions.
Health Checkup	Includes checkup records (Lab value, past medical history, hereditary conditions, lifestyle, etc., retrieved from survey questionnaires) of a health checkup examinee.
Medical Institution	Includes information of a medical institution (address, the number of hospital beds) attended by a health checkup examinee.
Senior Long-Term Care	Includes information on subjects’ application for long-term care services, usage records, and the status of facilities

**Table 4:** Features in health checkup cohort DB.

Table	Feature Code *	Feature Description	Purpose of Use
Common	STD_YYYY	Year between 2016 and 2019	Merging between tables
	RN_INDI	A six-digit code for interlinking the tables	
	RN_INST	A six-digit code for interlinking the tables	
Qualification	SEX	1: Male, 2: Female	Sociodemographic feature
	AGE	Patient’s age in a corresponding year	
	SIDO	City code	
	GAIBJA_TYPE	A code indicating the type of insurance	Patient feature
	CTRB	Income quantile (1–10)	
	DSB_SVRT_CD	No inclusion, severe, mild level of disability	
Birth and Death	DTH_YYYYMM	The date of one’s death	Dependent variable
Diagnosis	HSPTZ_PATH_TYPE	Admission route	Grouping
	MCARE_RSLT_TYPE	Patient’s condition on the day of one’s final treatment	Censoring
Medical Institution	INST_CLSFC_CD	Type of medical institutions	Institution feature
	CNT_DR_TOT	The number of doctors	
	CNT_NRS_TOT	The number of nurses	
	CNT_BED_INP	The number of hospital beds	
Medical Institution	CNT_BED_OP	The number of surgical beds	Institution feature
	CNT_BED_ER	The number of emergency beds	

* Feature code is directly retrieved from the data provided by NHIS.

**Table 5:** Preprocessed results by feature (Survival Time Determinant Analysis).

Category	Feature	Preprocessed Results
Sociodemographic Information	Gender	[Grouping] Male/Female
	Age	[Grouping] 10-year unit
	City	[Grouping] Seoul/Metropolitan city/Small and medium-sized city
Patient Information	Insurance status	[Grouping] Workplace/Regional/Medical insurees [Grouping] 0/1–3/4–7/8–10 [Grouping] No inclusion, severe, mild
	Income quantile	
	Severity of disability	
Institution Information	Institution type The number of doctors per 100 hospital beds	[Grouping] top general hospitals/general hospitals [Feature preprocessing] (the number of doctors/the number of hospital beds) × 100 [Grouping] in four quantiles [Feature preprocessing] (the number of nurses/the number of hospital beds) × 100 [Grouping] in four quantiles [Grouping] in four quantiles
	The number of nurses per 100 hospital beds	
	The number of hospital beds The number of surgical beds The number of emergency beds	

**Table 6:** Characteristics of general inpatients.

Category	Feature		Number of Patients	Number of Dead	Censored Data	
					N	%
Sociodemographic Characteristics	Gender	Male	1,618,558	493,753	1,124,805	69.49
		Female	1,610,375	362,935	1,247,440	77.46
	Age	~29	376,400	8,557	367,843	97.73
		30~39	181,316	11,059	170,257	93.9
		40~49	304,032	38,933	265,099	87.19
		50~59	548,986	97,958	451,028	82.16
		60~69	619,434	155,199	464,235	74.95
		70~79	684,980	261,576	423,404	61.81
		80~89	447,479	236,122	211,357	47.23
		90~	66,306	47,284	19,022	28.69
	City	Seoul	690,213	216,436	473,777	68.64
		Metropolitan city	1,011,287	240,263	771,024	76.24
		Small, medium-sized city	1,527,433	399,989	1,127,444	73.81
Patient Characteristics	Insurance status	Medical insuree	324,647	110,780	213,867	65.88
		Regional insurance	927,937	247,733	680,204	73.30
		Workplace insurance	1,976,349	498,175	1,478,174	74.79
	Income quantile	0 (= Medical aid)	324,647	110,780	213,867	65.88
		1~3	653,717	161,816	491,901	75.25
		4~7	1,014,811	238,137	776,674	76.53
		8~10	1,235,758	345,955	889,803	72.00
	Severity of Disability	Normal	2,543,100	596,918	1,946,182	76.53
		Mild	364,475	129,482	234,993	64.47
		Severe	321,358	130,288	191,070	59.46
Institution Characteristics	Type of medical institution	General hospital	2,256,811	521,261	1,735,550	76.9
		Top general hospital	972,122	335,427	636,695	65.5
	Number of doctors per 100 hospital beds	~13	851,638	164,396	687,242	80.7
		14~30	758,352	173,799	584,553	77.08
		31~46	841,977	271,496	570,481	67.75
		47~	776,966	246,997	529,969	68.21
	Number of nurses per 100 hospital beds	~52	803,234	167,841	635,393	79.1
		53~80	769,585	179,191	590,394	76.72
		81~103	846,852	245,625	601,227	71.00
		104~	809,262	264,031	545,231	67.37
	Number of hospital beds	~280	768,741	144,734	624,007	81.17
		~281~519	844,725	193,682	651,043	77.07
		520~749	806,039	238,870	567,169	70.36
		750~	809,428	279,402	530,026	65.48
	Number of surgical beds	~4	687,623	141,742	545,881	79.39
		5~9	925,752	190,039	735,713	79.47
		10~15	781,024	250,246	530,778	67.96
		16~	834,534	274,661	559,873	67.09
	Number of emergency beds	~15	776,801	146,626	630,175	81.12
		15~22	807,330	190,381	616,949	76.42
		23~35	830,467	250,014	580,453	69.89
		36~	814,335	269,667	544,668	66.89
Total			3,228,933	856,688	2,372,245	

**Table 7:** Characteristics of inpatients admitted through the Emergency Room.

Category	Feature		Number of Patients	Number of Dead	Censored Data	
					N	%
Sociodemographic Characteristics	Gender	Male	571,640	371,216	200,424	35.06
		Female	543,474	388,387	155,087	28.54
	Age	~29	143,679	139,950	3729	2.60
		30~39	63,773	59,087	4686	7.35
		40~49	93,932	81,225	12,707	13.53
		50~59	161,395	127,515	33,880	20.99
		60~69	186,934	129,766	57,168	30.58
		70~79	237,902	132,291	105,611	44.39
		80~89	194,696	81,129	113,567	58.33
		90~	32,803	8640	24,163	73.66
	City	Seoul	258,178	169,368	88,810	34.4
		Metropolitan city	284,779	195,224	89,555	31.45
		Small, medium-sized city	572,157	395,011	177,146	30.96
Patient Characteristics	Insurance status	Medical insuree	110,136	64,425	45,711	41.50
		Regional insurance	320,967	219,090	101,877	31.74
		Workplace insurance	684,011	476,088	207,923	30.4
	Income quantile	0 (=Medical aid)	110,136	64,425	45,711	41.50
		1~3	224,901	158,102	158,102	70.3
		4~7	341,334	247,235	247,235	72.43
		8~10	438,743	289,841	148,902	33.94
	Severity of Disability	Normal	865,904	622,833	242,261	27.98
		Mild	127,769	72,803	54,966	43.02
		Severe	122,251	63,967	58,284	47.68
Institution Characteristics	Type of medical institution	General hospital	684,519	487,287	197,232	28.81
		Top general hospital	430,595	272,316	158,279	36.76
	Number of doctors per 100 hospital beds	~13	135,227	96,104	39,123	28.93
		14~30	245,888	177,896	67,992	27.65
		31~46	398,310	263,256	135,054	33.91
		47~	335,689	222,347	113,342	33.76
	Number of nurses per 100 hospital beds	~52	139,881	97,734	42,147	30.13
		53~80	227,528	163,339	64,189	28.21
		81~103	390,697	267,089	123,608	31.64
		104~	357,008	231,441	125,567	35.17
	Number of hospital beds	~280	138,642	102,709	35,933	25.92
		~281~519	230,297	163,236	67,061	29.12
		520~749	393,323	269,569	123,754	31.46
		750~	352,852	224,089	128,763	36.49
	Number of surgical beds	~4	130,599	94,109	36,940	28.29
		5~9	243,815	179,372	64,443	26.43
		10~15	372,513	248,428	124,085	33.31
		16~	368,187	237,694	130,493	35.44
	Number of emergency beds	~15	115,294	81,769	33,525	29.08
		15~22	243,659	177,732	65,927	27.06
		23~35	370,612	249,681	120,931	32.63
		36~	385,549	250,421	135,128	35.05
Total			1,115,114	759,603	355,511	

**Table 8:** Determinants of the death rate (general inpatients vs. inpatients admitted through the ER).

Category	Feature	General inpatient	Inpatients Admitted through ER
Sociodemographic Characteristics	Gender	Male > Female	Male > Female
	Age	Above 90s > 80s > 70s > 60s > 50s > 40s > 30s > below 30s	Above 90s > 80s > 70s > 60s > 50s > 40s > 30s > below 30s
	City	Small and medium-sized city > Seoul > Metropolitan city	Metropolitan city > Small and medium-sized city > Seoul
Patient Characteristics	Insurance status	(Medical insurees = Regional insurance) > Workplace insurance	Medical insurees > Regional insurance > Workplace insurance
	Income quantile	4–7 quantiles > 1–3 quantiles >(0 quantile = 8–10 quantiles)	4–7 quantiles > 1–3 quantiles > (0 quantiles = 8–10 quantiles)
	Severity of disability	Normal > (Severe = Mild)	Normal > Mild > Severe
Institution Characteristics	Type of institution	Top general hospital > General hospital	Top general hospital > General hospital
	Number of doctors per 100 hospital beds	31–48 > 14–30 > above 47 > below 14	14–30 > 31–48 > (above 47 = below 14)
	Number of nurses per 100 hospital beds	Above 104 > 81–103 > 53–80 > below 53	(Below 53 = Above 104) > 53–80 > 81–103
	Number of hospital beds	Below 281 = 281–519 = 520–749 = above 750	281–519 > above 750 > (below 281 = 520–749)
	Number of surgical beds	(Below 5 = 10–15 = above 16) > 5–9	Above 16 > 10–15 > below 5 > 5–9
	Number of emergency beds	23–35 > above 36 > 15–22 > below 15	Above 36 > 23–35 > 15–22 > below 15

## Data Availability

Data sharing is not applicable to this article.
